# Computed tomographic angiography in planning thoraco-dorsal artery perforator flap in breast and soft tissue reconstruction: a systematic review

**DOI:** 10.1093/bjr/tqae203

**Published:** 2024-10-08

**Authors:** Mahmoud Soliman, Islam H Metwally, Adel Denewer, Ahmed Abdallah, Fatmaelzahraa Abdelfattah Denewar, Nuala Ann Healy, Laszlo Romics, Amit Agrawal

**Affiliations:** North Manchester General Hospital, Manchester University NHS Foundation Trust, Manchester, M8 5RB, United Kingdom; Surgical Oncology Department, Faculty of Medicine, Mansoura University, 35516, Egypt; Surgical Oncology Department, Faculty of Medicine, Mansoura University, 35516, Egypt; Surgical Oncology Department, Faculty of Medicine, Mansoura University, 35516, Egypt; Surgical Oncology Department, Faculty of Medicine, Mansoura University, 35516, Egypt; Radiology Department, Faculty of Medicine, Mansoura University, 35516, Egypt; Beaumont Breast Center, Beaumont Hospital, Dublin, D09V2N0, Ireland; Department of Radiology, Royal College of Surgeons, Dublin, D02 YN77, Ireland; Department of Surgery, Greater Glasgow & Clyde NHS (Gartnavel General Hospital & Royal Alexandria Hospital, Paisley), Glasgow, PA2 9PN, United Kingdom; Cambridge Breast Unit, Cambridge University Hospitals NHS Foundation Trust, Cambridge, CB2 0QQ, United Kingdom

**Keywords:** computed tomographic angiography, thoraco-dorsal artery perforator flap, thoraco-dorsal artery perforator mapping, breast and soft tissue reconstruction

## Abstract

**Objectives:**

Thoraco-dorsal artery perforator (TDAP) flaps have been increasingly used in breast and soft tissue reconstruction. Perforator localization is often done using a hand-held doppler, however, false results are not uncommon. This study aimed to systematically review the literature on the value of preoperative computed tomographic angiography (CTA) in TDAP flaps examining scanning protocol, mapping technique, concordance with operative findings, and disadvantages.

**Methods:**

A PRISMA-compliant comprehensive search of Medline, Embase, Cochrane Library, and CINAHL databases was conducted in November 2023. We included studies evaluating CTA mapping of free and pedicled TDAPs for breast or soft tissue reconstruction using The Joanna Briggs Institute (JBI) Critical Appraisal Tools.

**Results:**

Five studies were included and considered at high risk of bias. The studies included 72 patients with a mean age of 43.8 years. Concordance between CT findings and Doppler mapping or operative visualization was reported in two studies. In three studies, CTA was combined with Doppler flowmetry, whilst dynamic infrared thermography was used in one study. Standardized scanning protocol and patient positioning were lacking in all reports.

**Conclusions:**

This study highlights the paucity of evidence on the value of CTA in TDA perforator mapping with inconsistent outcomes and non-standardized scanning protocols. Despite difficult imaging acquisition and interpretation, 3D reconstructed images and detailed vascular anatomy may facilitate planning.

**Advances in knowledge:**

Further research is required to explore the practical value of CTA in TDAP planning and standardizing protocols.

## Introduction

The emerging concept of pedicled chest wall perforator flaps has pushed the boundaries of reconstructive surgery, reducing donor-site morbidity.^1–4^ The thoraco-dorsal artery perforator (TDAP) flap has been described in partial and total breast reconstruction, coverage of prosthetic implants, and as a salvage option.^5–8^ It was also reported in skin and soft tissue resurfacing in the chest wall, head, neck, and extremities.^9^ However, raising a perforator flap requires accurate anatomical knowledge and meticulous tissue handling. Image-assisted surgery provided further reliability of results, shorter operative time, and less complication rate.^10^

Although the hand-held Doppler is the current standard method of perforator localization, it may be inaccurate alongside insufficient functional information. Computed tomographic angiography (CTA), on the other hand, provides detailed anatomical information on the perforator vessels and can be reformatted into 3D images, facilitating surgical planning.

Meta-analyses and systematic reviews on the role of preoperative CTA in abdominal-based free flap reconstruction showed lower complication rates, shorter operative time, and higher perforator identification rates.^11^ However, there is a lack of data on its value in TDAP flap reconstruction.^11–13^

This systematic review aims to evaluate the role of preoperative CTA in mapping TDA perforators, exploring the CTA imaging protocol, location of perforators, number of perforators, and concordance between CTA mapping and operative findings.

## Methods

A comprehensive PRISMA-compliant search was conducted, including all studies published between 1946 and November 2023 in MEDLINE (PubMed), Embase (Ovid SP), Cochrane Library, and CINAHL (EBSCO) databases. This included relevant subject or MesH headings in addition to a manual assessment of reference lists in eligible full-text articles. Non-English language, anatomical/cadaveric studies, and narrative reviews were excluded.

The following sample search of all fields was used in all databases:

- CONCEPT (1): Tdap flap* OR Tap flap* OR Tapia flap* OR thoracodorsal artery perforator* OR thoracodorsal artery perforator flap* OR Thoraco-dorsal artery perforator* OR Thoraco-dorsal artery perforator flap* OR thoracodorsal perforator flap* OR subject/MeSH heading (thoracodorsal artery perforator flap/)
- CONCEPT (2): angiograph* OR computed tomograph* OR computer assisted tomograph* OR computer-assisted tomograph* OR computed tomographic angiography OR comput* tomogra* angio* OR ct angiography OR ct angio* OR subject/MeSH heading (computer assisted tomography/or computed tomographic angiography/or angiography/)
- Search (1) AND (2)

All studies evaluating CTA in the mapping of the TDA perforators for preoperative planning of TDAP flap reconstruction of the breast, extremity, or soft tissue were included. After removing duplicate studies, the first author (MS) screened the titles and abstracts of articles independently based on the inclusion criteria. The full text was obtained and assessed in case the title or abstract was unclear or relevant. The full text for abstracts that met the inclusion criteria (*n* = 17) was obtained and independently assessed for eligibility by two authors (MS, IH).

**Figure 1.**
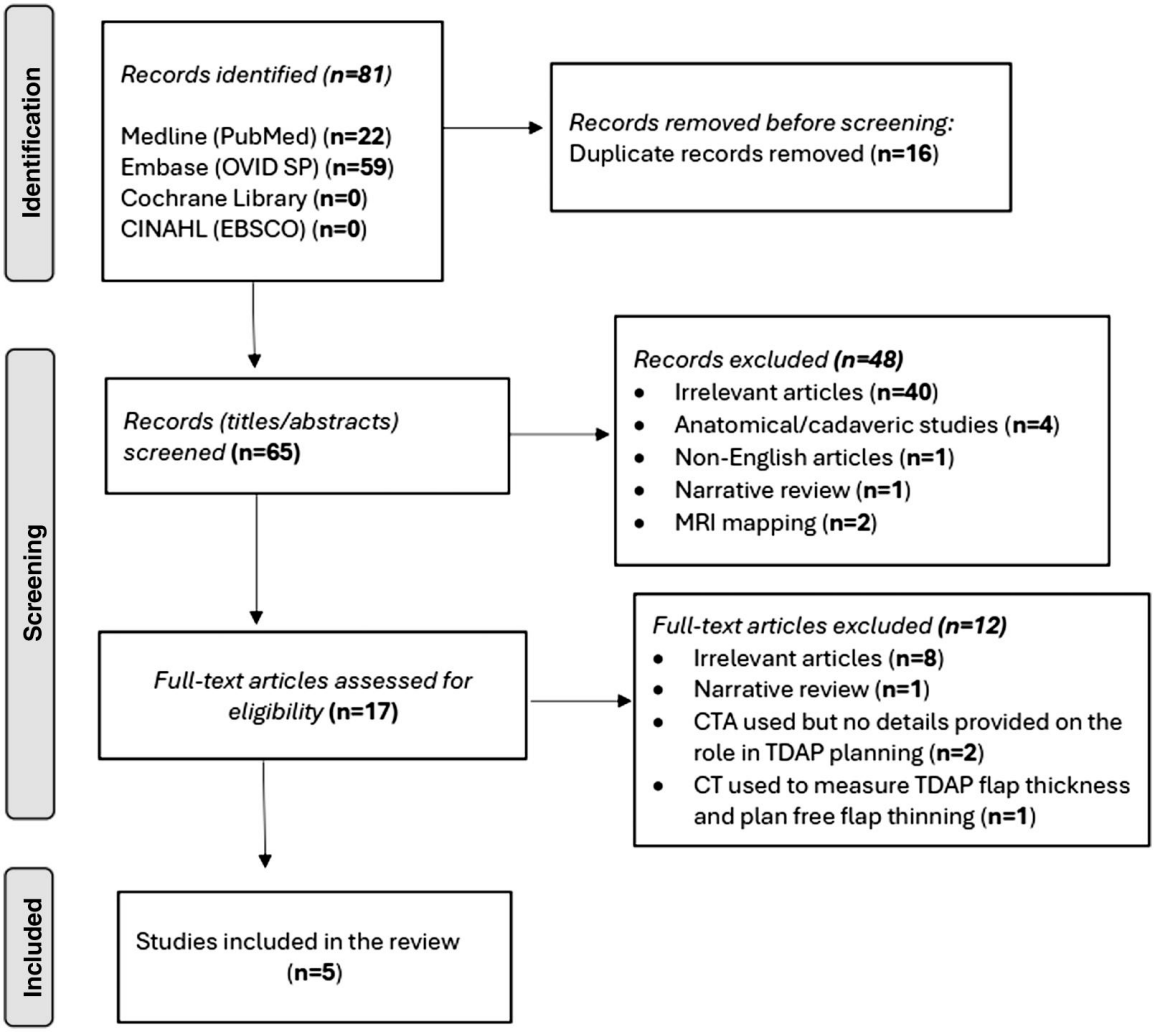
PRISMA diagram for the systematic review demonstrating the search results, included and excluded studies.

Data were extracted using a standardized Microsoft Excel™ spreadsheet. All study designs were included. Studies were excluded if they used the CT scan for another purpose in planning TDAP flaps, such as measuring flap thickness. Studies that described the use of CTA in TDAP planning met the inclusion criteria if any of the following was explained: scanning protocol, perforator mapping technique, or mapping outcomes. Scanning protocol and localization technique include information on contrast injection, scan initiation, patient positioning, and technicalities of perforator localization. Mapping outcomes include concordance with Doppler or intraoperative findings, the number and/or location of mapped perforators. A PRISMA flow chart showing the selection process is shown in Figure 1. Appraisal of the quality of evidence was done using The Joanna Briggs Institute (JBI) Critical Appraisal Tools for case reports, case series, and cohort studies.^14^

## Results

Five studies were included: one case report, three case series, and one retrospective cohort study (Table 1).^10^^,^^15–18^ Generally, there was significant heterogeneity among the included studies with lack of similarity of the measured outcomes preventing a meaningful analysis. Therefore, a narrative synthesis of the studies was performed, highlighting key findings, and data were summarized.

**Table 1.** Summary of the included studies.

	Study type	Patient (*n*)	Age (range)	Reconstruction	Flap type
Hamdi et al (2007)^15^	Case report	1 (female)	49	Breast	Pedicled flap
Mun et al (2008)^17^	Retrospective cohort (2005-2007)	25 (15 males, 10 females)	36.6 (7-65)	Extremities and head and neck	Free flap
Kim et al (2012)^16^	Retrospective case series (2010-2011)	19 (13 males, 6 females)	39.3 (10-75)	Extremities	Free flap
Ojeda et al (2013)^10^	Retrospective case series (2010-2011)	11 (females)	44.2 (28-63)	Breast	Pedicled flap
Sjøberg et al (2020)^18^	Prospective case series	16 (1 male, 15 females)	50 (21-65)	Breast and axilla	Pedicled flap
Total/mean (range)	Variable	72 (14 males, 32 females)	43.8 (7-75)	Variable	Variable

Although two studies referred to the use of CTA in localizing TDA perforators, they were excluded as no further information was provided to explain the exact role of CTA in the planning process, and without any details on the scanning technique, perforator mapping, or operative findings.^19^^,^^20^ One excluded study used CTA to measure the thickness of the TDAP flap but not for perforator localization.^21^

Only two studies specifically examined the role of CTA in mapping TDAP perforators reporting the concordance between CT and operative findings.^16^^,^^17^ Both studies were conducted on free TDAP flaps for soft tissue reconstruction of the extremities, head, and neck; only one demonstrated the location and number of CTA-mapped perforators.^17^ The remaining three studies did not report concordance or mapped perforators’ numbers or locations. Sjøberg et al^18^ mainly investigated the pre- and intraoperative use of dynamic infrared thermography (DIRT) in mapping TDAP perforators and assessing flap perfusion with complementary preoperative CTA. Hand-held Doppler was used as a complementary tool in three studies. Quality assessment of the studies is shown in three tables in the Supplementary Information.

### Computed tomographic angiography scanning protocol and patient positioning

**Figure 2.**
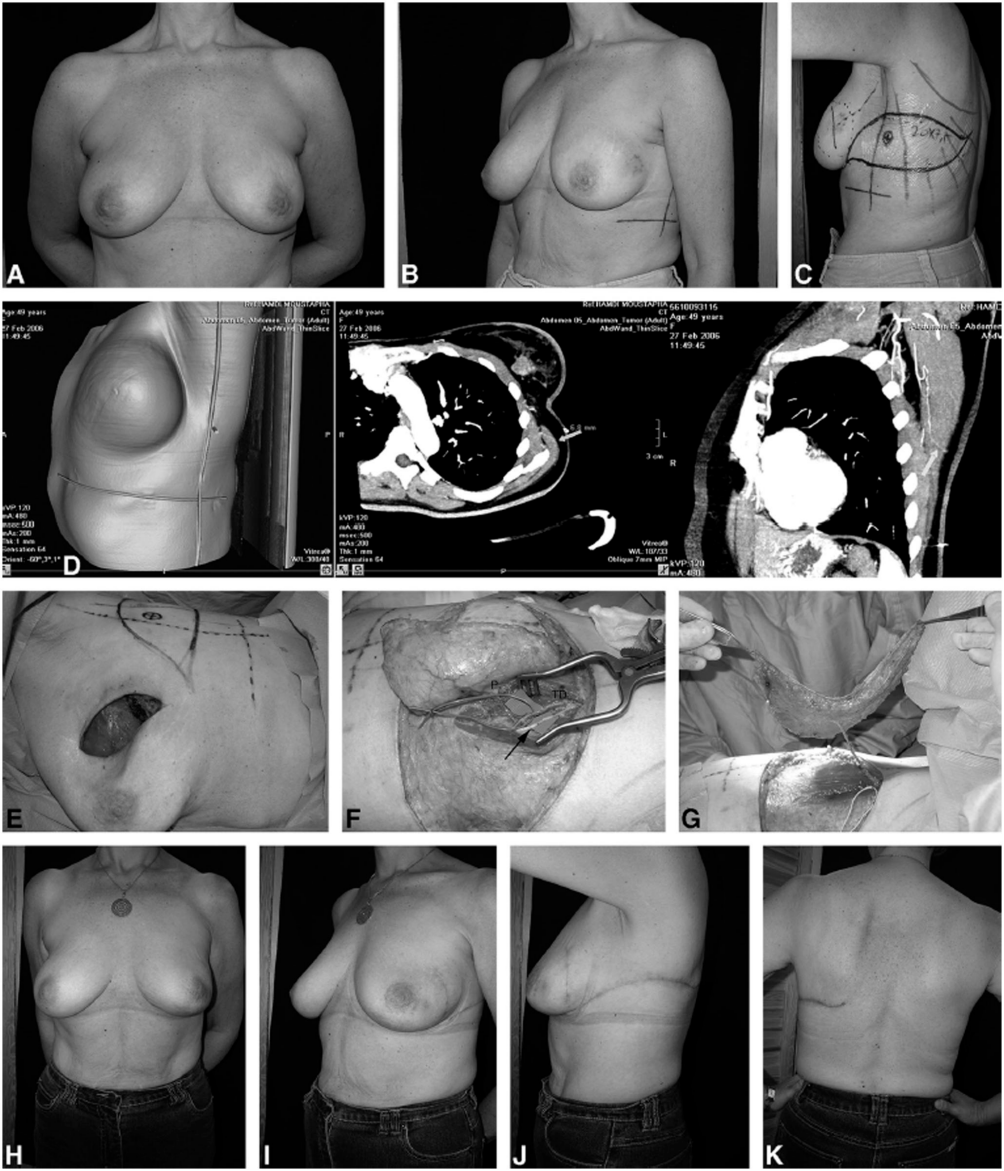
Hamdi et al demonstrated perforator identification on CTA and Volume Rendering (VR) and explained the application onto the patient. (A) A patient having partial breast reconstruction using a pedicled TDAP flap. (A, B) Preoperative views. (C) Marking the TDAP flap and the perforator (circled cross) guided by the CT. (D) CT images with the dominant perforator shown (arrow) on the sagittal and horizontal sections and transferred onto the patient’s 3D image. As a landmark, a lead cross was used on the patient's skin. (E) Resulting breast defect following wide local excision. (F) TDAP perforator dissection up to the thoraco-dorsal vessels (vessel loop) by splitting the LD muscle. Complete preservation of the thoraco-dorsal nerve (arrow). (G) Complete harvest of the TDAP flap. (H, I) Postoperative results at 3 months. (J, K) The donor site. The scar is hidden in the bra region. (used with permission of Wolters Kluwer Health, Inc.).^15^

Hamdi et al^15^ first used multidetector CT (MDCT) in planning TDAP flaps for immediate partial breast reconstruction. Although they generally outlined the protocol for CT imaging in different perforator flaps, it was unclear if the same protocol was used with TDAP planning. For accurate localization of the perforator, they applied a lead cross on the patient as a reference before imaging (*X*-axis at the lateral free border of LD and *Y*-axis below the breast) (Figure 2).

**Figure 3.**
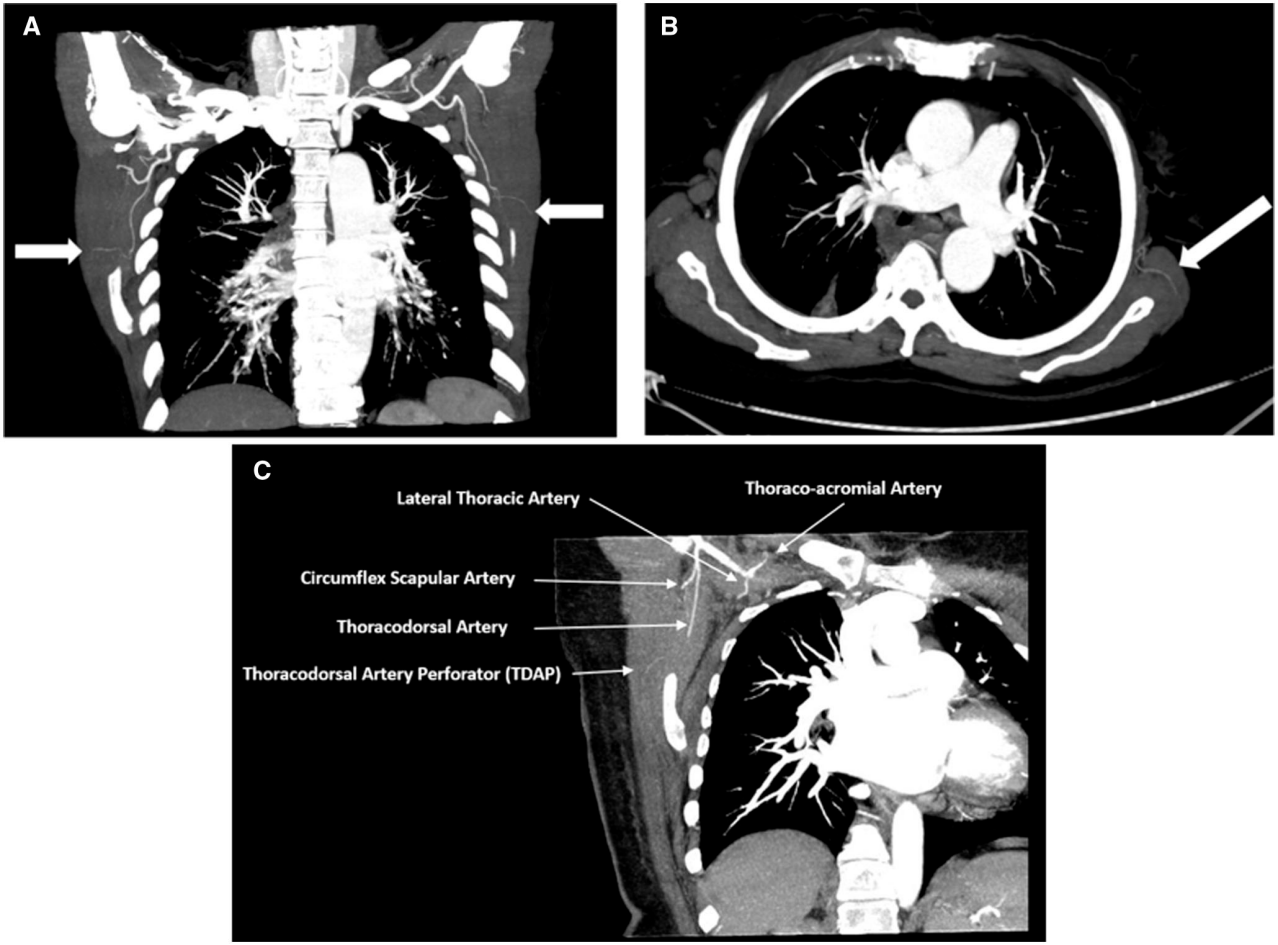
(A) CTA (maximum intensity projection, MIP) coronal view showing single TDA perforator identified bilaterally arising from the descending branch (white arrow); (B) Left TDA perforator on axial view (white arrow); (C) Branching pattern of the axillary artery: lateral thoracic artery and thoraco-acromial artery originating from the second part and subscapular artery from the third part giving off the circumflex scapular artery to continue as the TDA ending with TDA perforator.

In general, the scanning protocol aims to achieve the arterial phase, eliminate venous contamination, and maximize arterial filling. Therefore, the scan should be triggered from the pedicle’s origin following the direction of blood flow through the perforators. When scanning for deep inferior epigastric artery perforator (DIEP) flaps, the scan is initiated from the external iliac/common femoral artery junction and in a caudocranial direction. Rozen et al^22^ emphasized that perforators can be visualized using a routine CTA of the chest. However, a further 5-second delay between the opacification of arch of the aorta and the start of the scan is often required allowing the contrast to fill the perforators completely (Figure 3). Different CT scanning protocols and technical considerations in the included studies are demonstrated in Table 2.

**Table 2.** CT scanning protocol and patient positioning.

	Detector-row	Contrast injection and flow rate	Scan initiation (bolus tracking)	Scanning range	Positioning	Slice thickness (mm)	Slice interval (mm)
Hamdi et al (2007)^15^	64	120 mL (400 mg/mL) at 4 mL/s	15 s after the enhancement of the aortic arch reaching the preferred point	N/A	Supine with shoulder extension	1	0.6
Mun et al (2008)^17^	16	120 mL (non-ionic contrast, 300 mg/mL) at 3-4 mL/s	3 s after the enhancement of the aortic arch reaching the preferred point (100 Hounsfield Unit)	Shoulder to D10	Lateral position + shoulder extension 100°-120°	1.25	1.25
Kim et al (2012)^16^	320	150 mL (non-ionic contrast, 300 mg/mL) at 3 mL/s	N/A	Shoulder to the umbilicus	Lateral or supine + shoulder extension	1	0.8
Ojeda et al (2013)^10^	64	120 mL (non-ionic contrast, 300 mg/mL) at 4 mL/s	N/A	Cervical area to the diaphragm	Supine	N/A	N/A
Sjøberg et al (2020)^18^	128	80 mL (350 mg/mL) at 4 mL/s	>100 Hounsfield Unit (HU) at aortic arch with 5 s delay	Clavicle to xiphoid process	Supine with shoulder extension	0.6	0.4

### Outcomes of preoperative CT angiography for TDAP perforator mapping

Mapping outcomes are summarized in Table 3. In a series by Kim and colleagues,^16^ CTA identified TDAP perforators in all 19 cases. The descending branch of the TDA had large reliable perforators in 14 patients, whilst six patients had their perforators arising from the transverse branch, and one had perforators from both branches. Perforators originating from the descending branch were easier to find in the coronal view, whilst perforators from the transverse branch were only detected in the axial view. However, examining both planes provided information on the intramuscular course of the perforators, facilitating muscular dissection and shortening operative time.

**Table 3.** TDAP perforator mapping outcome and conclusions.

	Patients who had perforators identified (*n*)	Was the number of mapped perforators reported?	Was complementary handheld doppler used?	Was concordance reported?	Conclusion
Hamdi et al (2007)^15^	1/1	No	No	No	CTA mapping recommended especially in bilateral reconstruction
Mun et al (2008)^17^	25/25	Yes	Yes^a^	Yes	CTA mapping recommended
Kim et al (2012)^16^	19/19	No	No	Yes	CTA mapping recommended
Ojeda et al (2013)^10^	N/A	No	Yes	No	N/A
Sjøberg et al (2020)^18^	N/A^b^	No	Yes	No	CTA mapping not recommended

Abbreviations: CTA = computed tomographic angiography; N/A = not available.

a All perforators marked using CTA produced an audible sound on the Doppler examination.

b The continuation of the perforator into the subcutaneous tissue layer was only seen in one patient despite the visualization of the intramuscular perforators in many patients.

The reconstructed 3D images provided a practical, visually applicable tool for surgeons to identify perforator locations, and the study recommended the use of coronal and axial views with 3D reconstruction, preferably with 1 mm slice thickness and intervals of 0.8 mm to reduce the chance of missing perforators.

**Figure 4.**
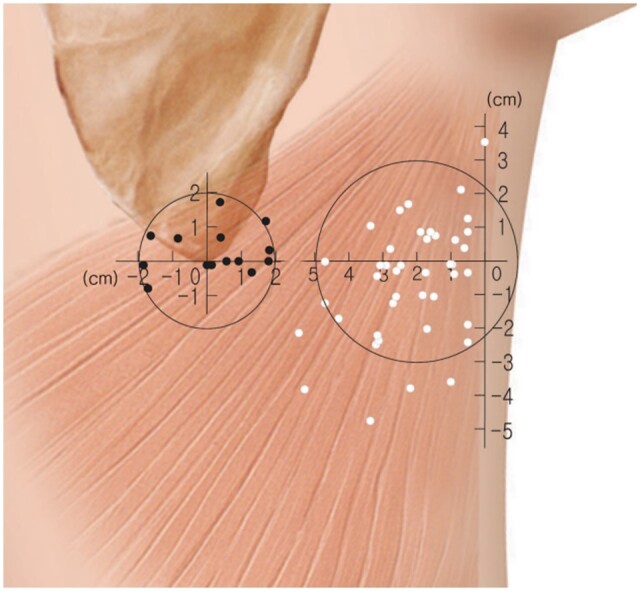
Illustration by Mun et al showing the location of the marked perforators; 85.7% of the lateral perforators (white dots) were located within a 3-cm-radius circle with the centre of the circle marked at a point 2 cm medial to the lateral border of the muscle and at the level of the inferior angle of scapula. Among medial perforators (black dots), 92.8% were in a 2-cm-radius circle centred at the inferior scapular angle (used with permission of Wolters Kluwer Health, Inc.).^17^

**Figure 5.**
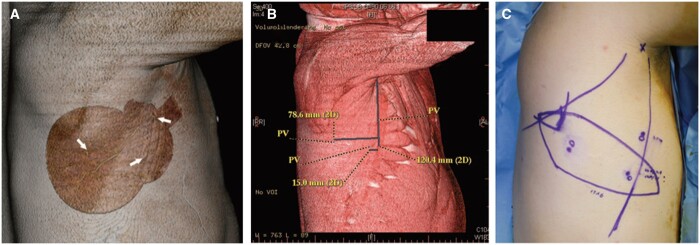
Perforator localization technique as shown by Mun et al. (A) Three localized perforators (arrows); (B) Perforators visualized in the volume-rendered image; (C) Mapped perforators were translocated to the patient and the flap was designed and positioned appropriately. Dots represent CT-marked perforators and open circles represent the Doppler-based perforator locations (used with permission of Wolters Kluwer Health, Inc.).^17^

Mun et al^17^ identified 1-4 (mean 2.2) TDA perforators. Of 56 marked perforators, 37 perforators were found within 4 cm of the lateral border of the LD muscle, and 14 perforators within 4 cm of the medial border. About 85.7% of the lateral perforators were located within a 3-cm-radius circle with the centre of the circle marked at a point 2 cm medial to the lateral border of the muscle and at the level of the inferior angle of scapula. The level of the scapular inferior angle was distal to the origin of the posterior axillary fold by 9.8 cm (range 5.3 to 12.5 cm). Likewise, among medial perforators, 92.8% were in a 2-cm-radius circle centred at the inferior scapular angle. These two topographic-defined zones can be explored initially intra-operatively for the identification of a perforator (Figure 4). CTA-localized points showed a good concordance with Doppler examination within 15 mm distance (mean 7.1 mm) (Figure 5).

They also retrospectively compared the surgical outcomes of 25 patients with the same procedure without prior CT planning. The flap harvest time was significantly shorter in the CT mapping group (77.2 vs 101.0 min, *P* = .01). Further, in seven patients who had small TDAP flap reconstruction (<50 cm^2^), CT mapping was associated with significantly shorter donor-site scar compared to no CT mapping (172.4 vs 126.6 mm, *P* = .03). In the two groups, age, body mass index, rate of flap failure, flap size, and the number of perforators used were similar. The importance of reproducing the patient’s position during CT was also brought to attention; the patients had the CT in the same position as during surgery.

Ojeda et al^18^ reported using volume rendering and maximum intensity projections in 11 out of 22 cases to localize the perforators with the largest calibre and assess whether the perforator was routeing intra-muscular or inter-fascial. Further information on mapping success rate and concordance with surgical findings was not discussed.^10^ In a series mainly evaluating DIRT in TDAP planning, even though CT angiography identified the TDA, its branching pattern and the intramuscular perforators in all patients, the continuation of an intramuscular perforator into the subcutaneous layer was only detected in one patient. A summary of the advantages and disadvantages of preoperative CT angiography in TDAP flap planning (extrapolated from the included studies) is shown in Table 4.

**Table 4.** CT angiography in TDAP perforator mapping: advantages and disadvantages.

Advantages	Disadvantages
Quick examination	Contrast-related complications
Identifying dominant TDAP perforator	Radiation exposure
Identify alternative perforators, eg, LTAP	Difficult reproducibility
Helpful in cases with previous axillary surgery	Challenging interpretation
Evaluation of several potential donor sites simultaneously	Need for radiographer/technician training
Faster complementary doppler mapping and decreased operative time	Increased cost
Assessment of subcutaneous tissue thickness and facilitating free flap thinning	Incidental CT findings

Abbreviations: CT = computed tomographic; LTAP = lateral thoracic artery perforator; TDAP, thoraco-dorsal artery perforator.

## Discussion

Our systematic review shows the scarcity of data on the use and cost-effectiveness of CT angiography in TDAP flap reconstruction. We only identified five studies where the CTA was used for mapping TDA perforators; only two of them reported the concordance between CT findings and Doppler mapping or operative visualization.^16^^,^^17^ The number and location of perforators localized using CTA were only reported in one study by Mun et al. Main advantage for CTA was the detailed anatomical and functional information and surgeon-friendly 3D reconstructed images.

Surgical planning for perforator flaps can only depend on the knowledge of the anatomical location of perforators without preoperative imaging. Still, unexpected anatomical variations can be encountered, adding to the complexity of perforator selection and flap design and potentially lengthening the flap harvest time.^23^ Hand-held Doppler is the most popular method for perforator localization because of its simplicity, accessibility, and much lower cost.^24^ However, it can have false results and provide less anatomical and functional information.^25^

In a recent systematic review on partial breast reconstruction using a TDAP flap, most authors performed preoperative mapping of perforator vessels using a Doppler probe (six studies), whilst colour Doppler ultrasonography was used in one study, and only one study reported the use of a 3D CTA.^26^ In a narrative review, CTA localized the dominant TDA perforator and evaluated other potential donor sites allowing flexibility in the donor site and perforator selection. Reconstructed 3D images ensured a smooth transition from the scans to the real-life markings.^22^

Our study demonstrates the lack of a standardized scanning technique and patient positioning among studies. Boucher and colleagues^27^ proposed a standardized CT protocol with an intravenous contrast concentration of 350-400 mg/mL at a flow rate of 4 mL/s followed by 30 mL of saline injection. It was also emphasized that the patient should be positioned as planned on the operating table during image acquisition, which will be a lateral position with arm abduction with TDAP flap.

MDCT seems to be accurate based on the identification of TDA perforators in all 44 patients where mapping outcomes were reported,^16^^,^^17^ nevertheless, it has not been utilized by most surgeons due to limitations such as small perforators, difficult acquisition and interpretation of images, and radiation exposure. Mun et al^17^ reported that interpreting CT images in back donors was more challenging than interpreting abdominal flaps, mainly because of the small size of the perforators.

CT angiography can be associated with more concerns regarding radiation exposure in breast cancer patients.^28^ Iodinated contrast, additionally, may be linked to small but serious risks of anaphylaxis and nephrotoxicity.^29^ In one study, CTA was found to be limited in detailing the subcutaneous course of the perforator, subsequently hindering the mapping process.^18^ This observation underlines that identifying the full course of the TDA perforators is not as straightforward as in abdominal perforators.

In DIEP flaps, incidental findings were found to increase the actual cost of CTA to as high as 30%. Of note, there was approximately a 5-fold increase in incidental findings by adding chest imaging to CTA of the abdomen to assess for internal mammary and/or thoraco-dorsal artery patency.^30^ Yet, evidence on cost-effectiveness concerning the TDAP flap is lacking.

In the anatomical study by Kim and colleagues,^21^ CT imaging was used to investigate the subcutaneous tissue layer in the TDA territory. In free perforator flap surgery, complex flap designs such as chimeric and dual-paddle flaps can be confidently planned with the help of CTA. This, combined with delineating the intramuscular course of the perforator, the appraisal of recipient vessels and timesaving in bilateral reconstructions, may point to the fact that angiography may be of more value in planning free rather than pedicled TDAP flaps.^17^

In a retrospective study reporting the use of free TDAP flaps for large defects (>20 cm), CTA-guided identification of multiple TDA perforators including the dominant one was found to marginally improve flap survival compared to single perforator-based large TDAP flaps. Careful mapping also avoided switching to the muscle-sparing LD flap design by incorporating a strip of muscle, which, although safer and easier, is more restricting when flap thinning is required.^19^

Perforator mapping using magnetic resonance angiography (MRA) was mainly reported in abdominal-based flaps with the advantage of no radiation exposure, less incidence of contrast allergy, and negligible risk of renal insufficiency.^31^^,^^32^ Vasile and colleagues^33^ reported the use of MRA in TDAP localization.

Our review has several limitations, including inconsistent data reporting and methodology across small studies with no clear pre-defined outcomes. The type of reconstruction (pedicled vs free), the indication of reconstruction, and the scanning protocol were variable among the included studies, with one study describing the number and location of mapped perforators. One underlying factor common to all studies’ limitations is the limited early experience in the surgical technique and, hence, associated imaging techniques.

Within this study’s limitations, the authors believe there is no sufficient evidence to recommend routine preoperative CTA for localizing TDA perforators. Nevertheless, evidence suggests that CTA could be of more value in planning free TDAP flap. Of note, TDA perforators may be simultaneously localized when performing staging CT for oncologic indications, if indicated. This study highlights areas of potential future research on the use of CTA in TDAP reconstruction, including standardizing the CTA scanning technique, preoperative perforator localization, and cost-effectiveness of CTA. In early breast cancer patients, the increase in breast MRI for staging of breast tumours may enable simultaneous assessment of perforators if reconstruction is planned.

## Conclusions

Our study highlights the paucity of evidence on the value of CTA in TDA perforator mapping. Despite difficult imaging acquisition and interpretation, 3D reconstructed images, and detailed intramuscular anatomy may facilitate surgical planning especially in free TDAP flap reconstruction.

## Supplementary Material

tqae203_Supplementary_Data
