# Supplementary material for: Computed tomographic angiography in planning thoraco-dorsal artery perforator flap in breast and soft tissue reconstruction: a systematic review
Source: Br J Radiol. 2024 Oct 8;98(1165):27–35. doi: 10.1093/bjr/tqae203 (PMC11652726; doi:10.1093/bjr/tqae203)
Supplement: tqae203_Supplementary_Data [file tqae203_supplementary_data.docx]

**Table 1:** Joanna Briggs Institute (JBI) critical appraisal checklist for case reports. A judgment of the risk of bias is shown.

| Author | **Hamdi et al (2007) [16]** |
| --- | --- |
| Were patient’s demographic characteristics clearly described? | **High** risk |
| Was the patient’s history clearly described and presented as a timeline? | Low risk |
| Was the current clinical condition of the patient on presentation clearly described? | Low risk |
| Were diagnostic tests or assessment methods and the results clearly described? | **High** risk |
| Was the intervention(s) or treatment procedure(s) clearly described? | Low risk |
| Was the post-intervention clinical condition clearly described? | Low risk |
| Were adverse events (harms) or unanticipated events identified and described? | *Unclear* |
| Does the case report provide takeaway lessons? | Low risk |

**Table 2:** Joanna Briggs Institute (JBI) critical appraisal checklist for case series. A judgment of the risk of bias is shown.

| Author | **Kim et al (2012) [17]** | **Sjøberg et al (2020) [19]** | **Ojeda et al (2013) [10]** |
| --- | --- | --- | --- |
| Were there clear criteria for inclusion in the case series? | Low risk | **High** risk | Low risk |
| Was the condition measured in a standard, reliable way for all participants included in the case series? | Low risk | Low risk | **High** risk |
| Were valid methods used for identification of the condition for all participants included in the case series? | **High** risk | *Unclear* | *Unclear* |
| Did the case series have consecutive inclusion of participants? | Low risk | *Unclear* | **High** risk |
| Did the case series have complete inclusion of participants? | *Unclear* | *Unclear* | **High** risk |
| Was there clear reporting of the demographics of the participants in the study? | Low risk | Low risk | Low risk |
| Was there clear reporting of clinical information of the participants? | Low risk | Low risk | Low risk |
| Were the outcomes or follow up results of cases clearly reported? | Low risk | Low risk | **High** risk |
| Was there clear reporting of the presenting site(s)/clinic(s) demographic information? | *Unclear* | *Unclear* | *Unclear* |
| Was statistical analysis appropriate? | *Unclear* | *Unclear* | *Unclear* |

**Table 3:** Joanna Briggs Institute (JBI) critical appraisal checklist for cohort studies. A judgment of the risk of bias is shown.

| Author | **Mun et al (2008) [18]** |
| --- | --- |
| Were the two groups similar and recruited from the same population? | Low risk |
| Were the exposures measured similarly to assign people to both exposed and unexposed groups? | Low risk |
| Was the exposure measured in a valid and reliable way? | Low risk |
| Were confounding factors identified? | **High** risk |
| Were strategies to deal with confounding factors stated? | **High** risk |
| Were the groups/participants free of the outcome at the start of the study (or at the moment of exposure)? | Low risk |
| Were the outcomes measured in a valid and reliable way? | Low risk |
| Was the follow up time reported and sufficient to be long enough for outcomes to occur? | *Unclear* |
| Was follow up complete, and if not, were the reasons to loss to follow up described and explored? | *Unclear* |
| Were strategies to address incomplete follow up utilized? | *Unclear* |
| Was appropriate statistical analysis used? | Low risk |
